# Split-Hopkinson Pressure Bar Testing of Water with Partial Lateral Confinement

**DOI:** 10.1007/s11340-024-01134-1

**Published:** 2025-01-07

**Authors:** K.S.O. Li, A. Van Lerberghe, A. D. Barr, A. A. Dennis, S. D. Clarke

**Affiliations:** Department of Civil & Structural Engineering, University of Sheffield, Mappin Street, Sheffield, S1 3JD UK

**Keywords:** High-strain-rate testing, Split-Hopkinson pressure bar, Partial lateral confinement, LS-DYNA, SPH, Water

## Abstract

**Background:**

For the first time, the high-strain-rate behaviour of water is investigated experimentally and validated to LS-DYNA numerical simulations, using Smooth Particle Hydrodynamics (SPH).

**Objective:**

This paper presents the application of a modified split-Hopkinson pressure bar (SHPB) fitted with a partial lateral confinement apparatus on a water specimen.

**Method:**

The lateral confinement is provided by a water reservoir surrounding the specimen. A pressure transducer is installed in the reservoir wall to measure lateral stresses, and a dispersion correction algorithm, SHPB_Processing.py, is utilised to obtain accurate measurements of axial and radial stresses and strains.

**Results:**

Experimental results underscore the capability of the modified apparatus to assess triaxial behaviour of water under high-strain rates. Comparisons with numerical modelling reveal that cohesion between water particles is non-existent, highlighting an intrinsic limitation in numerical modelling.

**Conclusion:**

These results highlight the capability to perform characterisation of fluids under high-strain rates. While limitations in numerical modelling still exist, numerical modelling and experimental testing using the modified apparatus can be applied to characterise fluid behaviour in the future.

## Introduction

The split Hopkinson pressure bar (SHPB) is a common tool used for characterising the behaviour of materials under high strain-rate conditions, ranging from $10^2$ s$^{-1}$ to $10^4$ s$^{-1}$. Soils testing employing the SHPB are commonly performed by confining a soil specimen in a rigid tube or ring, limiting lateral displacement. These uniaxial strain experiments are effective for characterising soil compaction response at different strain rates [1–3], as well as comparing soils with different moisture contents [3, 4], initial densities [5, 6] and particle size distributions [7], but have never been used to characterise the behaviour of liquids.

**Fig. 1:**
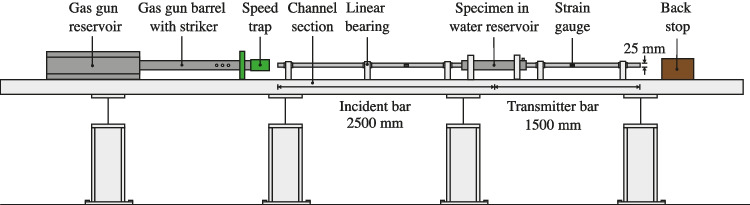
Schematic of the partially confined SHPB apparatus: Bar and reservoir configuration

Several authors have developed methods that allow lateral confinement to alter throughout a SHPB test to generate a triaxial stress state. Pierce and Charlie [8] used a steel tube lined with a membrane to investigate the wave speed of partially saturated sands, at varying confining stresses of 0 kPa and 310 kPa. While the steel tube prevented lateral strains from developing, water pressure applied between the tube and membrane provided additional confining stress, which was also transmitted along the pressure bars via a piston assembly on the transmitter bar. Bailly et al. [9] employed brass confining rings to imitate approximately elastic (near perfectly plastic) behaviour at high strain rates. The material specimen would initially be laterally confined within the rings and deform in uniaxial strain until the radial stress reached the yield point in the ring, at which point the specimen would begin to laterally deform at a quasi-constant confining stress.

Other authors have modified the traditional triaxial cell (CTC) for high-strain-rate testing. Christensen et al. [10] used a large pressure vessel to conduct triaxial tests on sandstone to confining stresses of 207 MPa. The specimen and pressure bars were enclosed in the pressure vessel, which had a hole at one end to facilitate loading of the incident bar, which was secured with a collar. Frew et al. [11] improved the triaxial SHPB further by incorporating pressure vessels around both the specimen and transmitter bar ends, allowing hydrostatic loading to be followed by a high strain rate deviatoric phase. This modified apparatus was utilised by Martin et al. [12] to test the shear response of sand at confining stresses between 25 MPa and 150 MPa, as well as strain rates of 500 s-1 and 1000 s-1.

Barr et al. [13] pioneered a modified SHPB experiment setup involving a partial lateral confinement reservoir that allows a confining stress to build passively during high-strain-rate axial loading. This method combines aspects of unconfined SHPB experiments (usually with a thin membrane) and fully confined SHPB experiments (often with a steel ring) to provide a more comprehensive picture of soil behaviour during high-strain-rate events. This is especially pertinent to blast and impact events, as research into the strain rate dependent behaviour of soils exhibited during high-strain-rates prompts its application in buried explosive scenarios.

The current work seeks to utilise the SHPB set up pioneered by Barr et al. [13] to investigate high-strain-rate effects of liquids, specifically water. This paper will investigate the high-strain-rate effects of water through the employment of the modified SHPB with partial lateral confinement.

## Experimental Setup

**Fig. 2:**
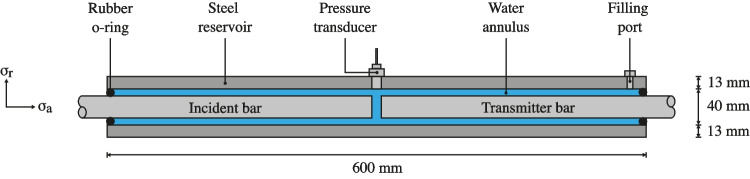
Schematic of the partially confined SHPB apparatus: water reservoir section with axial/radial axis convention

The modified SHPB is made up of a standard pressure bar arrangement which consists of a striker, an incident and a transmitter bar, 25 mm in diameter, with a 350 mm, 2500 mm and 1500 mm length, respectively, as shown in Fig. 1. In addition, as illustrated in Fig. 2, a 600 mm long steel water reservoir is set on linear bearings and centred around the specimen. When the pressure bars are in place, the annular gap present throughout the length of the reservoir is filled with water at atmospheric pressure, as depicted in Fig. 2.

**Fig. 3:**
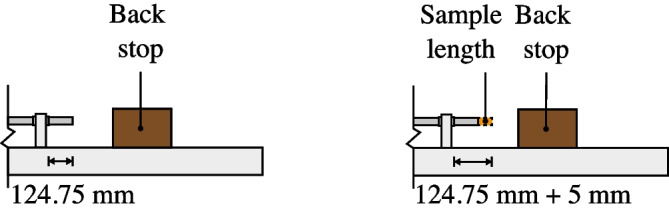
Schematic of sample measurements process before and after installation inside reservoir

The sample tested is water, therefore the entire reservoir is filled with water, and the radial stress response, $\sigma _r$, is measured by a pressure transducer mounted on the reservoir’s wall. While a rubber confinement method could also be used to restrict fluid in between the pressure bars, it would prevent radial pressure from being monitored. The axial stress response, $\sigma _a$, is measured with Kyowa KSP-2-120-E4 semiconductor strain gauges on the pressure bars, set up in pairs for the Wheatstone bridge arrangement.

The reservoir length was designed so that the time required for a stress wave initiated at the specimen surface to travel to and from the reservoir’s end exceeds the loading duration in the specimen, guaranteeing that inward-travelling waves from the boundary do not interfere with pressure measurements [13]. This simplifies the seal between the reservoir and the pressure bars, which are only needed to keep the water at atmospheric pressure.

## Sample Methodology

The application of this testing method was carried out on water to illustrate the capacity of the partially-confined SHPB and to validate that the chosen design results in reliable fluid pressure measurements. The water density tested was 1.0 Mg m$^{-3}$. Preparation of the sample was as follows:

1. Supports were installed on the channel around the incident and transmitter bars of the SHPB setup, prior to installing the steel reservoir providing lateral confinement for the sample.
2. The incident bar was placed into position, approximately 5 mm from the transmitter bar, this was measured as the change in length between the end of the transmitter bar and the final support. It was checked again before all supports were bolted down, and the test launched.
3. The water reservoir is translated into the centre of the setup, and the pressure port is aligned with the centre of the specimen.
4. The incident bar’s linear bearing, closest to the steel reservoir, is re-adjusted to its initial test position and bolted back down.
5. O-rings were inserted on either side of the water reservoir to seal its ends.
6. The reservoir was filled with water using a filling port and sealed by fitting the pressure transducer and filling port bolt. The transducer used in the experiment was a Kulite HKM-375-2500, calibrated by the manufacturer to perform linearly to a pressure of 25 MPa.
7. Measurement of the length between the two Hopkinson pressure bars was done one last time between the end of the transmitter bar and the final support (Fig. 3).

The method was carried out in the same manner as a standard SHPB experiment. Loading was done by striking the incident bar with a stainless-steel striker bar fired from a gas gun, at varying velocities. Tests were conducted at 16 m/s and 20 m/s, where speeds were recorded using a speed trap placed at the exit of the gas gun barrel.

Signals from the pressure bar strain gauges and pressure transducer were recorded using a TiePie Handyscope four-channel digital oscilloscope using 14-bit A-D resolution and a sample frequency of 1 MHz, with a record length of 131.072 kSa.

**Fig. 4:**
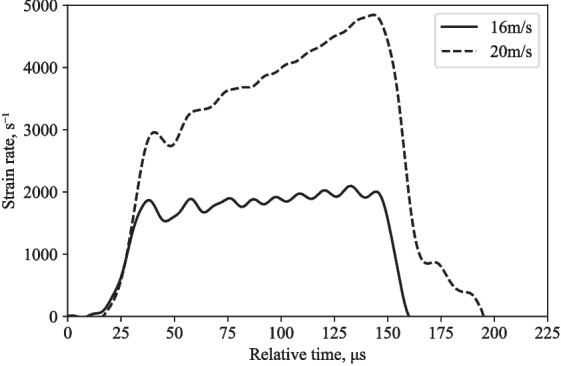
Variation of strain rate during partially-confined SHPB experiments on water

From these tests, conducted at two different speeds, a broad range of strain rate was captured, as shown in Fig. 4, where the strain rate increases to 2095 s$^-1$ and 4844 s$^-1$, over approximately 150 $\mu$s. Under these high-strain-rate conditions, both the axial and radial stresses of the specimen were measured.

## Signal processing

**Fig. 5:**
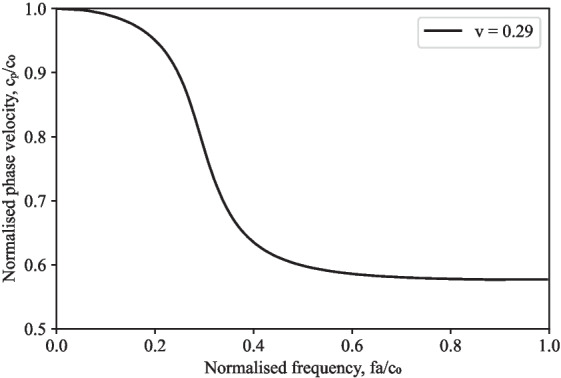
Phase velocity frequency relationship for the first mode of propagation of a longitudinal wave [3]

When processing signals from SHPB experiments, it is frequently believed that longitudinal stress waves in the pressure bars travel one-dimensionally at a common velocity $c_0$, and hence measurements recorded at strain gauges are frequently simply translated to the end of the bar using a suitable time delay [14]. In actuality, stress waves travel at a certain phase velocity, $c_p$, which is a function of frequency, bar diameter, one-dimensional wave speed and Poisson’s ratio [15], as illustrated in Fig. 5 [3].

As the frequency of a wave grows, the phase velocity drops, resulting in signal dispersion as it propagates down the bar. The dispersion of the stress pulse is accompanied by a frequency-dependent variation in stress and strain across the bar cross-section, so a signal recorded on the surface of the bar at some distance from the specimen will not accurately reflect the stresses the specimen was subjected to, and therefore cannot be used to accurately determine specimen response.

The pressure bar signals were processed using an open-source Python algorithm, SHPB_Processing.py, with specific functionalities for partial lateral confinement testing using SHPB setups [16]. It uses an implementation of Tyas and Pope’s dispersion-correction approach via a subroutine titled dispersion.py, to verify that the inferred measures of axial stress and strain appropriately depict the specimen behaviour [17]. In this script the method utilised is as follows:

1. Fast Fourier transform (FFT) is used to transfer the time-domain strain signal to the frequency domain.
2. To account for the dispersion over the distance between the strain gauge and the bar end, the phase angle of each frequency component is corrected using the relationship illustrated in Fig. 5.
3. The amplitude of each frequency component is corrected using the factors M1 and M2, which account for the fluctuation of strain and Young’s modulus over the bar cross section, respectively. These are derived from Davies’ analysis of the radial effects in a cylindrical pressure bar [18].
4. Using the inverse FFT, the signal is then converted back into the time domain.

The dispersion adjustment is especially crucial in determining the stress transmitted into the specimen from the incident bar since it is determined from the sum of the incident and reflected waves, both of which contain considerable high-frequency components.

**Fig. 6:**
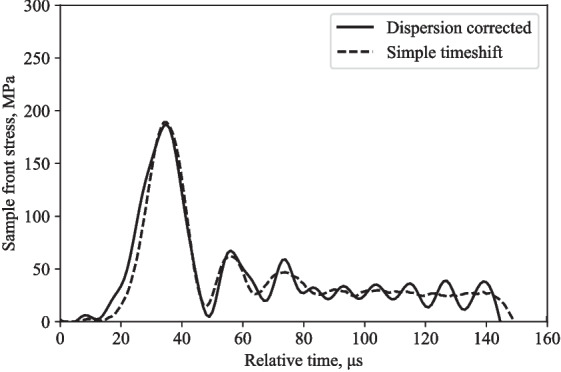
Partially confined SHPB test on water: front stress computed using dispersion correction and simple timeshifting

The incident and reflected stress waves measured at the incident bar strain gauge are assumed to maintain their shape as they are translated along the time axis using simple timeshifting whereas in the corrected method the dispersion associated with 1000 mm travel in the bar is added to the incident wave and removed from the reflected wave.

Figure 6 shows how the dispersion-corrected approach minimises the amplitude of the stress wave and eliminates an initial fluctuation in stress, which would have led to incorrect inferences about the specimen’s behaviour. In this case, dispersion effects are minimal.

**Fig. 7:**
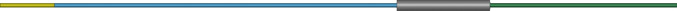
LS-DYNA SHPB partial lateral confinement model set up

**Fig. 8:**
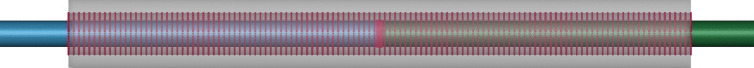
LS-DYNA cross section zoom-in on the sample inside the partial lateral confinement SHPB set up

## Numerical Modelling

### Model Setup

The numerical modelisation of the arrangement in Fig. 7 was carried out using the explicit finite element code in LS-DYNA [19], in order to compare numerical and experimental results. A more detailed representation of the water sample and confinement reservoir are shown in Fig. 8. The model was created in 3D, where the striker bar (yellow in Fig. 7), incident bar (blue in Fig. 7) transmitter bar (green in Fig. 7) and steel reservoir (grey in Fig. 7) were modelled as Lagrangian solid mesh. SPH node modelisation was used to model the water sample (red in Fig. 8) [20–23].

For simplicity, the steel reservoir is modelled as a rigid steel boundary material, assuming that the fluid pressures generated will not be large enough to cause significant radial strains in the reservoir. The rubber rings were replaced with a boundary constraint to prevent the water from exiting the reservoir from the ends.

Automatic nodes-to-surface contact were selected for contact representation between the water sample made with SPH nodes and the lagrangian members of the incident and transmitter bars. Automatic nodes-to-surface contact was also utilised between the water sample and the steel reservoir. Manual surface-to-surface contact adjustments were made between lagrangian members in the model, such as between the striker and incident bars, and between the incident and transmitter bars.

### Model material cards

The three steel pressure (striker, incident and transmitter) bars were modelled as linear elastic materials (*MAT_ELASTIC) with a density, Young’s modulus and Poisson’s ratio of $\rho$ = 7850 kg m$^{-3}$, *E* = 168 GPa, $\nu$ = 0.29 respectively based on existing known properties of steel.

For all analyses, to match the experimental tests conducted, the striker bar was given an initial impact velocity of 16 m/s or 20 m/s similar to match the speeds tested experimentally. The steel reservoir was modelled as rigid (*MAT_RIGID), with a density, Young’s modulus and Poisson’s ratio of $\rho$ = 7850 kg m$^{-3}$, *E* = 168 GPa, $\nu$ = 0.29 respectively. The SPH water sample that encompassed the water annulus and the gap between the pressure bars was modelled using the linear polynomial equation of state (EOS):

1 $$\begin{aligned} \begin{aligned} P = C_0 + C_1 \mu + C_2 \mu ^2 + C_3 \mu ^3 \\ + (C_4 + C_5 \mu + C_6 \mu ^2)E \end{aligned} \end{aligned}$$

where $C_0$, $C_1$, $C_2$, $C_3$, $C_4$, $C_5$ and $C_6$ are constants, $\mu$ = $\rho$/$\rho _0$ - 1, $\rho$ and $\rho _0$ are the current and initial densities of the fluid, and *E* is the specific internal energy of the fluid. Table 1, displays the properties used to apply the null material card (*MAT_NULL), which only requires density input, and equation of state parameters utilised in this work for water. The constants for the equation of state were based on previous work by Shin [20], which studied the use of numerical modelling to simulate shock wave effects in water. To assign the initial pressure of the water to be equal to atmospheric pressure (101kPa), the specific internal energy, $E_0$, was found by applying eq. (1) with the constants from Table 1 to be 205.36kPa.

**Table 1:** Material model and equation of state (EOS) parameters for water (SI units) [20]

MAT_NULL							
1000							
EOS_LINEAR_POLYNOMIAL							
$C_0$	$C_1$	$C_2$	$C_3$	$C_4$	$C_5$	$C_6$	$E_9$
0.0	2.190E9	9.224E9	8.767E9	0.4934	1.3937	0.0	205.36E3

## Results

**Fig. 9:**
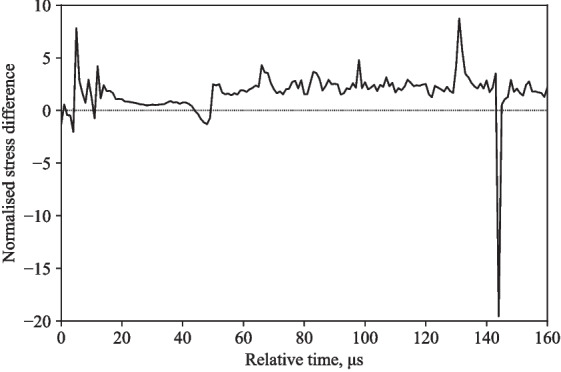
Typical response of a partially confined SHPB test on water showing axial and radial stress difference normalised by their mean

Figure 9 display the typical stress difference between axial and radial stress, illustrating the viability of the current configuration in assisting with the triaxial response of a liquid. The near zero stress difference indicates the translation of axial stress into radial stress when subject to loading, a property that aligns with the Poisson’s ratio of water.

**Fig. 10:**
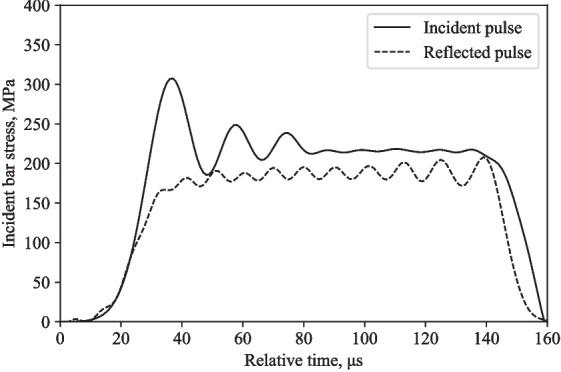
Typical behaviour of a partially confined SHPB experimental test on water at 16 m/s: incident and reflected pulses from the incident bar

**Fig. 11:**
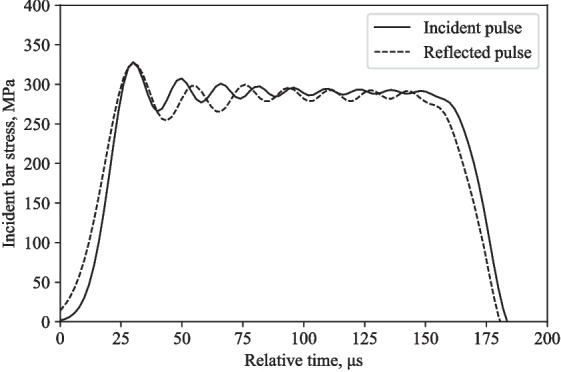
Typical behaviour of a partially confined SHPB LS-DYNA model on water at 16 m/s: incident and reflected pulses from the incident bar

Figures 10 and 11 show that the experimental and numerical incident pulses have the same amplitude at the same gauge locations, but the reflected pulses are very different.

**Fig. 12:**
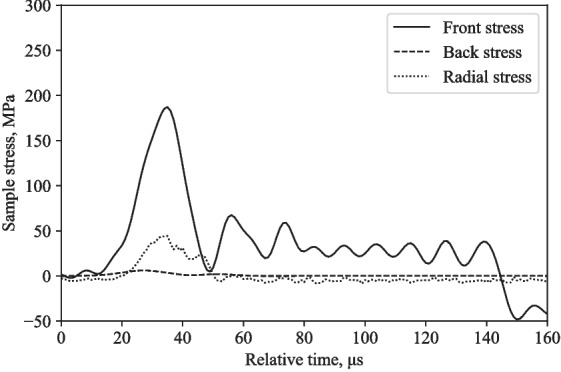
Partially confined SHPB test on water at 16 m/s: front, back and radial stresses

Tests were performed using the modified SHPB fitted with the partial lateral confinement reservoir on water, at 16 m/s and 20 m/s. Figures 12 and 14 show similarities in terms of response behaviour, with a logical increase in amplitude associated with its higher test speed.

Figures 12 and 14 depict the experimentally measured front, back and radial stresses. The radial stress directly adjacent to the water in between the pressure bars was calculated by taking into account the transit time of the radial stress wave through the water annulus (5.1 $\mu$s, assuming a wave speed in water of 1482 m/s). The recorded radial stress shows a radial stress wave with peaks that align relatively well with front and back stresses, indicating that the lateral response recorded with the pressure transducer is a direct result of the axial loading from the SHPB test.

Looking at the Poisson’s ratio, experimentally at 16 m/s, the maximum front, back and radial stress recorded are 180, 5 and 46 MPa, respectively, resulting in a Poisson’s ratio of 0.5 (Fig. 12), when the axial stress ([180 + 5]/2 = 92.5 MPa) is divided by the radial stress (46 MPa). Since the theoretical Poisson’s ratio for water is 0.5, this indicates that the axial and radial stress data obtained by employing this modified SHPB setup exhibit a degree of accuracy reflected in theory.

At higher striker speeds, the incident bar’s inertia and the partial lateral confinement steel reservoir will have an impact on the front, back and radial stresses, as seen in Fig. 14. This will have an effect on the Poisson’s ratio of the water specimen, progressively lowering its value.

**Fig. 13:**
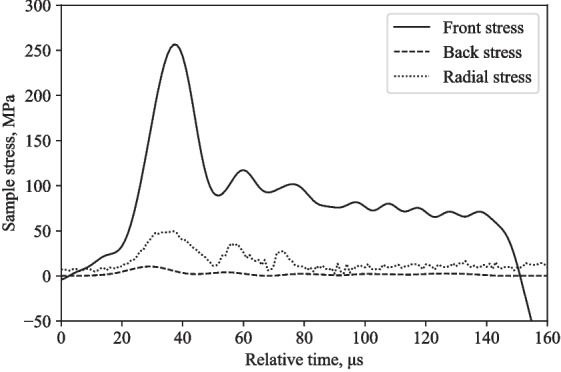
Partially confined SHPB LS-DYNA model on water at 16 m/s: front, back and radial stresses

**Fig. 14:**
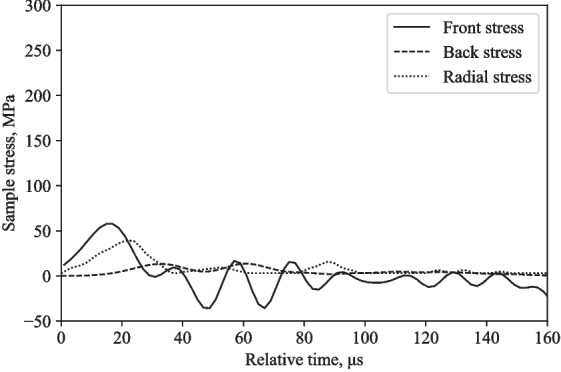
Partially confined SHPB test on water at 20 m/s: front, back and radial stresses

**Fig. 15:**
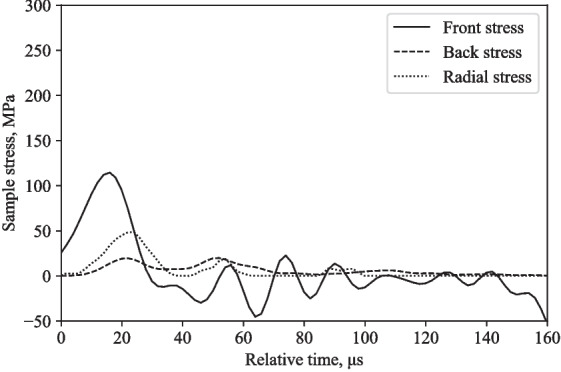
Partially confined SHPB LS-DYNA model on water at 20 m/s: front, back and radial stresses

The back stress values differ by 60-80 %, radial stress values differ by 11-13 %, and the front stress values differ by 76-105 %, when comparing numerical and experimental stresses at 16 m/s and 20 m/s (Figs. 12, 13, 14, and 15).

While there is evidently a distinct disparity between the stress magnitudes from experimental and numerical results, numerical modelling is used in conjunction with experimental results to provide commentary on the viability of the apparatus in investigating stress behaviour of water. As such, qualitative and quantitative comparison between numerical and experimental results still exhibit similarities in terms of stress propagation tendencies through the fluid medium.

## Discussion

The capabilities of the modified SHPB with the implementation of the lateral confinement apparatus allow for high-strain-rate testing on water to explore its axial and lateral responses. The results collected from the experimental tests and numerical model in LS-DYNA, revealed a significant difference in front stress, leading to a considerable lower axial stress. This is due to the numerical model’s lack of cohesion between SPH particles when modelling a fluid like water, as evidenced by the two key arguments below:

1. The water sample in the modified SHPB with the lateral confinement apparatus, was modelled in LS-DYNA using SPH. This was done in the same manner as other projects that have modelled water for blast and high impact tests [21]. They used *MAT_NULL and the EOS linear polynomial, as shown in Table 1. However, *MAT_NULL only uses the initial density of the fluid. It does not include any cohesion parameters, which is a fundamental property of fluids. Water itself is a highly cohesive material. Consequently, omitting this will have a considerable impact on the numerical results.
2. When comparing modelling and experimental test results, the radial stress is within 10-13 % of the experimental values obtained, however, the back and front stresses are more than 60-80 % and 75-105 % away, respectively, from what it should be, experimentally. The radial stress is measured with a pressure transducer, while the front and back stresses are measured at the strain gauge location on the incident and transmitter bars in the model. Hence, the value obtained at these points is from the stress wave as it propagates through the SPH water particles, and hits the transmitter bar interface and pressure transducer. The significant difference in front and back stresses is due to the instant extrusion of the water sample upon contact from the incident pulse.

There is no cohesion between the SPH particles. The particles are instantaneously displaced in both horizontal and vertical directions, due to the impact of the stress wave considerably changing the size and shape of the specimen. There is no medium for the wave to propagate through. As a result, the water sample is unable to compact sufficiently to let the stress wave propagate through before extruding from in-between the Hopkinson pressure bars.

When comparing experimental and model outputs, it is evident the model is under predicting the stress results. This indicates that adding cohesion properties to this model would intuitively improve the specimen’s ability to withstand the stress wave passing through it.

Material cards that consider cohesion in LS-DYNA include *MAT_PSEUDO_TENSOR, *MAT_CONCRETE_DAMAGE, *MAT_FHWA_SOIL, *MAT_MOHR_COULOMB, *MAT_DRUCKER_PRAGER and *MAT_JOINTED_ROCK. However, the material cards *MAT_PSEUDO_TENSOR, *MAT_CONCRETE_DAMAGE, *MAT_FHWA_SOIL and *MAT_JOINTED_ROCK can not be used since they are made for steel, concrete, rock and soils, with some requiring an EOS and other parameters which could not be obtained for water. The material cards *MAT_MOHR_COULOMB and *MAT_DRUCKER_PRAGER had obtainable parameters, but showed the same behaviour as *MAT_NULL.

SPH parameters were explored in LS-DYNA, and it was discovered that there was no option to change the cohesion parameter for fluid modelling. Viscosity was evaluated in the numerical model and showed no effect on improving SPH particle cohesiveness, as it simply slowed their lateral and transverse movements.

The ability to evaluate the high-strain-rate behaviour of liquids and record both their lateral and axial stress responses fills a gap in present research that previously restricted SHPB testing to fluid materials.

Also, since high-strain testing on water can be directly used to compare the effect of saturation and actual water, the influence of water content on other materials such as soils can be better understood. The specific effect of soil parameters such as particle size or density can be examined more thoroughly by comparing high-strain and quasi-static triaxial tests on fully saturated soils.

While shear thickening fluids have not been explicitly investigated through this study, the opportunity to perform controlled high-strain-rate tests on specific fluids opens up the future opportunity to examine the ability of shear thickening fluids to dissipate energy. Adjustments to the numerical model including the modification of fluid medium viscosity would be crucial in indicating behaviour under various shear conditions. Shear thickening fluids are widely used in impact protection applications such as in shock absorbers or enhancing Kevlar fabrics, the implications that this apparatus provides would be vital in its further characterisation under high-strain-rate conditions [24, 25].

## Conclusion

An innovative testing methodology for partially-confined SHPB experiments has been used to test water at high-strain-rates, where the specimen is contained in a long sleeve reservoir. A pressure transducer in the wall of the reservoir is used to measure the radial stress of the specimen.

Experimental results showed a clear correlation between the increase of the strain rate and the amplitude of the radial and axial stresses. To compare with the experimental data collected from the tests, LS-DYNA numerical modelling of tests with and SPH water sample was undertaken. Although radial and back stresses were measured and represented in the numerical model with reasonable accuracy, substantial modelling constraints were discovered when looking at the front stress obtained from the model. This was due to a failure to account for the cohesion qualities of the SPH particles in the numerical model, which fluids naturally have.

The material card *MAT_NULL, which is commonly used to depict water in LS-DYNA, only requires its initial density; however, this material card does not account for the highly cohesive properties of water particles, an intrinsic property of fluids.

As a result, improvements to the existing model are required, such as creating a new material card in LS-DYNA that incorporates cohesion as a parameter for fluids and upgrading the modelling representation of SPH to account for cohesion between particles.

Experimentally, in addition to its capabilities for testing soils, this apparatus can be used to accurately characterise liquid materials at high strain-rates, which was previously impossible.

Future test series using this new apparatus will aim to define strain rate dependency as well as further investigate the influence of radial inertia observed in current tests. Furthermore, the results of high strain-rate water characterisation can be utilised to characterise very fine, undrained, fully saturated soils under high strain rate conditions.

## Data Availability

Data from this paper is available upon request from the corresponding author, Li K. S. O., at ksoli1@sheffield.ac.uk or li.oswald@gmail.com.
